# Crosstalk between lactate and tumor-associated immune cells: clinical relevance and insight

**DOI:** 10.3389/fonc.2024.1506849

**Published:** 2024-11-29

**Authors:** Kemin Sun, Ye Shen, Xiang Xiao, Hao Xu, Quanli Zhang, Ming Li

**Affiliations:** ^1^ Department of Thoracic Surgery, the Affiliated Cancer Hospital of Nanjing Medical University & Jiangsu Cancer Hospital & Jiangsu Institute of Cancer Research, Jiangsu Key Laboratory of Molecular and Translational Cancer Research, Collaborative Innovation Center for Cancer Personalized Medicine, Nanjing, Jiangsu, China; ^2^ The Fourth School of Clinical Medicine, Nanjing Medical University, Nanjing, Jiangsu, China; ^3^ School of Traditional Chinese Medicine, China Pharmaceutical University, Nanjing, China; ^4^ Department of Scientific Research, Jiangsu Cancer Hospital & the Affiliated Cancer Hospital of Nanjing Medical University & Jiangsu Institute of Cancer Research, Jiangsu Key Laboratory of Molecular and Translational Cancer Research, Nanjing, Jiangsu, China; ^5^ Department of Clinical Pharmacy, China Pharmaceutical University, Nanjing, China

**Keywords:** lactate, tumor microenvironment, Warburg effect, immunosuppression, cancer immunotherapy

## Abstract

Lactate, which was traditionally viewed as a metabolic byproduct of anaerobic glycolysis, has emerged as a significant signaling molecule involved in the development of tumors. Current studies highlight its dual function, where it not only fuels tumor development but also modulates immune responses. Lactate has an effect on various tumor-associated immune cells, promoting immunosuppressive conditions that facilitate tumor growth and immune evasion. This phenomenon is strongly associated with the Warburg effect, a metabolic shift observed in many cancers that favors glycolysis over oxidative phosphorylation, resulting in elevated lactate production. Exploring the complex interplay between lactate metabolism and tumor immunity provides a novel understanding regarding the mechanisms of tumor immune evasion and resistance to therapies. This review discusses the unique biology of lactate in the TME, its impact on immune cell dynamics, and its potential as a tumor treatment target.

## 1 Introduction

The recognition of lactate as a metabolic waste with harmful effects produced by cells under hypoxic conditions has evolved in recent years (1). A century ago, Otto Warburg first proposed the aerobic glycolysis phenomenon: tumor cells rapidly produce energy by glycolysis instead of oxidative phosphorylation (OXPHOS), even when there is an ample supply of oxygen. The phenomenon, which later acquired recognition as the “Warburg effect (2), has transformed lactate from a mere byproduct of metabolism to a signaling molecule that regulates metabolism, immune response, and intercellular communication (3). Both *in vitro* and *in vivo* experiments have demonstrated that the addition of lactate promotes tumor progression and treatment resistance (4). Moreover, metabolites such as lactate can act as acylase substrates or cofactors for epigenetic modifications (5). A 2019 study demonstrated the crucial role of lactate in promoting histone lysine residue modification. Resembling other posttranslational modifications (PTMs), lactylation regulates gene transcription and plays a significant part in inflammation and cancer (6). There is growing evidence that lactate has a profound impact on the growth progression, resistance to treatment, and immune evasion of tumors.

Tumors do not merely consist of abnormally proliferating cells but rather exhibit a highly structured system. The various components that make up a tumor are jointly known as the tumor microenvironment (TME) (7). In the context of TME, every element comprising the immune system is collectively referred to as the tumor immune microenvironment (TIME) due to their intricate interplay and crucial roles in tumor biology (8–10). Immune checkpoints (ICPs) are employed by cancer cells to evade immune system attacks (11). Now, immune checkpoint inhibitors (ICIs), like anti-cellular toxicity T lymphocyte-associated protein 4 (CTLA-4), anti-programmed death protein (PD-1), and anti-PD-1 ligand (PD-L1), have shown great promise in numerous cancer immunotherapies (12–14). Increasing evidence indicates that TIME exerts a more pivotal role in tumor immunity compared with ICPs (15–17). As the fundamental constituents of TIME, immune cells make crucial contributions to tumor immune responses. Distinct subsets of immune cells exhibit diverse functionalities and characteristics, making it essential to explore the functions of various immune cells in order to conquer cancers.

Tumor metabolic reprogramming, such as enhanced nutrient utilization, heightened oxygen uptake, and the reproduction of reactive nitrogen and oxygen species, can have a profound impact on immune responses (1–3). Furthermore, numerous metabolites present in the TME can influence the development and functional roles of immune cells (4, 5). Given the Warburg effect, it is reasonable to expect that the significantly elevated lactate concentration in the TME greatly influences the immune cells. Lactate can serve as a metabolic bridge between tumor cells and immune cells, facilitating the tumor’s enhanced adaptation to the microenvironment and evasion of immune surveillance. This review focuses on lactate within the TME and summarizes the distinct lactate metabolism observed in tumors. We analyze the effects of lactate on tumor-associated immune cells and investigate its clinical significance in the TME. like prognostic markers of tumors and potential drugs targeting lactate generation, transport, and lactylation for tumor immunotherapy.

## 2 Lactate biology in the TME

### 2.1 Special lactate metabolism

Pyruvate dehydrogenase (PDH) catalyzes the conversion of pyruvate from glucose into acetyl-CoA in the mitochondria during aerobic respiration (18, 19). As a result, acetyl-CoA enters the cycle of tricarboxylic acid (TCA) for OXPHOS. Through this process, each glucose molecule can generate 36 molecules of adenosine triphosphate (ATP) (20, 21). In periods of intense exercise and infection, when cells have an inadequate oxygen supply, pyruvate molecules do not enter the TCA cycle, but instead, cytoplasmic lactate dehydrogenase (LDH) catalyzes them to lactic acid. This metabolic pathway is commonly referred to as glycolysis (22, 23). Glycolysis functions as the principal pathway for the production of lactate, yet it exhibits lower efficiency in terms of energy generation compared to OXPHOS, resulting in a yield of only 2 ATP molecules per glucose molecule (24). Therefore, in aerobic conditions, normal cells tend to opt for OXPHOS, which yields higher energy production. Only under hypoxic conditions do they resort to the inefficient glycolysis. Lactate accumulation in the human body poses a significant risk due to its potential to cause lactic acidosis. Therefore, it is crucial to rapidly remove lactate from tissues and the circulatory system (25). Lactate converts to pyruvate before entering the mitochondria, where PDH facilitates its metabolization via the TCA cycle. Additionally, hepatic and muscular tissues can activate gluconeogenesis in response to lactate accumulation, converting it into glucose and releasing it into circulation for enhanced glucose utilization during energy expenditure (26).

**Figure 1:**
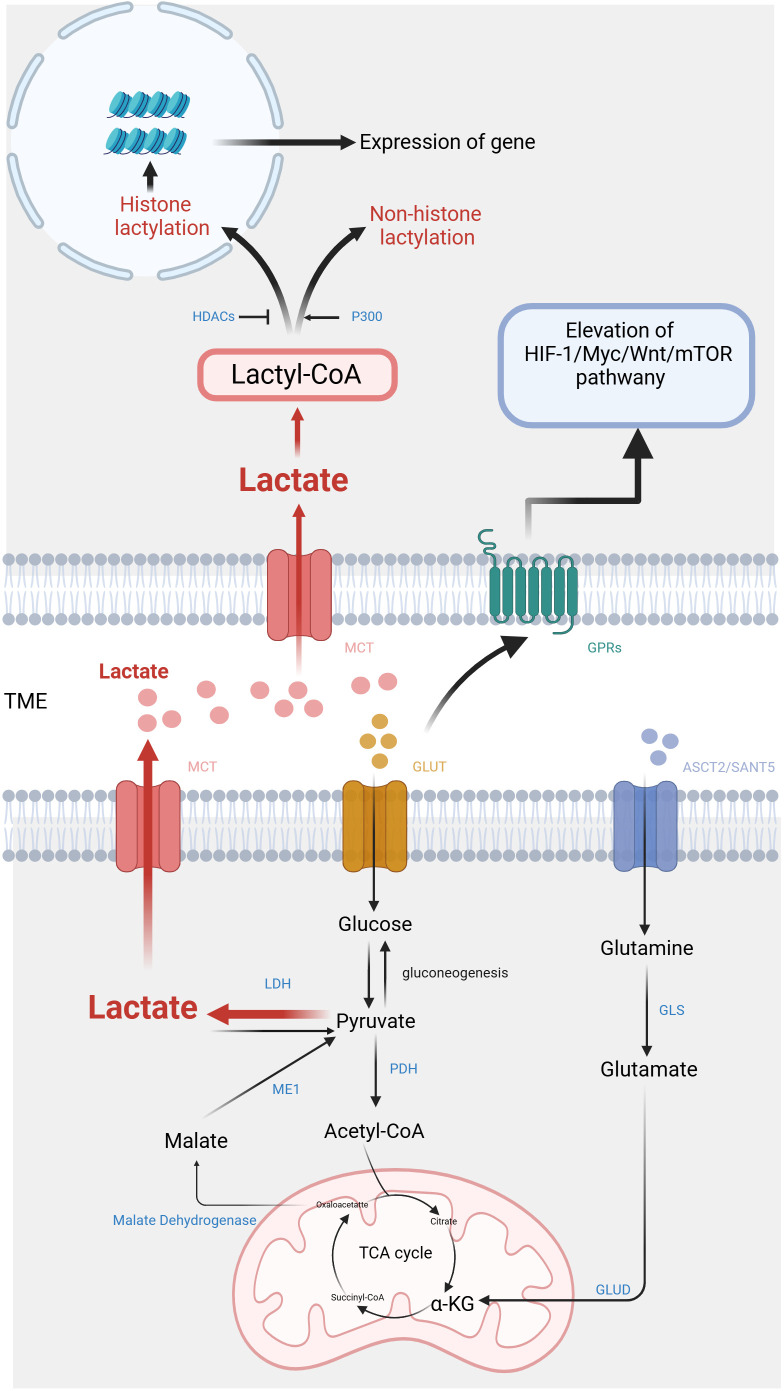
Metabolic pathways and signaling mechanisms of lactate in the tumor microenvironment. On one hand, lactate is produced by tumor cells through anaerobic glycolysis and is secreted into the extracellular space, where it contributes to the acidic microenvironment, promoting tumor progression and immune evasion. On the other hand, lactate also activates key signaling pathways, including the activation of HIF-1α, mTOR, and various inflammatory cytokines, which further influence tumor growth and metastasis. (Created with BioRender.com).

Malignant cells, as opposed to normal cells, exhibit a propensity for rapid energy generation through glycolysis despite the presence of sufficient oxygen (**Figure 1**). It is believed that tumor cells require this metabolic reprogramming to fulfill their energy requirements for growth and differentiation. Although strong glycolysis has been widely observed in tumor cells, the specific reasons and mechanisms behind it remain incompletely understood. However, research indicates that hypoxic cancer cells frequently demonstrate the activation of c-Myc and HIF-1α, resulting in enhanced anaerobic oxidation and increased lactate production (27–30). Pyruvate is transformed into lactate within the cell through LDH catalysis. The LDH protein is a heteromeric complex consisting of LDHA and LDHB, which exist in five isoforms. LDH-5 (A4) exhibits higher attraction for pyruvate than for lactate. Conversely, LDH-1 (B4) demonstrates a greater preference for lactate over pyruvate. Both c-Myc and HIF-1α upregulate LDH-5 activity and downregulate LDH-1 expression, thereby promoting lactate production (31). In summary, aerobic glycolysis can promote lactate production and lead to elevated concentrations of lactate in the TME.

Except for glycolysis, glutamine catabolism constitutes an alternative metabolic pathway employed by cancer cells for the lactate generation (32). Glutamine serves as a carbon framework for lactate production within the cancer cells (**Figure 1**). Glutamine is transported into the cell under the regulation of c-Myc, utilizing amino acid transporter protein type 2 (ASCT2) and sodium-coupled neutral amino acid transporter protein (SNAT5). Inside the cell, glutamine is converted to glutamate through the action of glutaminase (GLS). Subsequently, glutamate is transformed into α-ketoglutarate (α-KG) by glutamate dehydrogenase (GLUD) or various transaminases, including glutamate-oxaloacetate transaminase (GOT), glutamate-pyruvate transaminase (GPT), and phosphoserine aminotransferase (PSAT). α-KG then enters the TCA cycle. Within this cycle, carbon derived from glutamine is converted to oxaloacetate, which then exits the mitochondria to be transformed into malate. In the cytoplasm, malate undergoes further conversion into NADPH and pyruvate through the activity of malic enzyme 1 (ME1) (33). NADPH serves as essential during the biosynthesis of lipids and steroids, while pyruvate serves as a precursor for lactate. Reinforcing c-Myc activation can stimulate the glutamine metabolism, resulting in lactate production. This process establishes a positive loop that contributes to the accumulation of lactate (34).

### 2.2 Lactate shuttle and TME acidification

Lactate shuttle denotes the complete process of transmembrane lactic acid migration (6), which serves as the primary mechanism of lactate entering and exiting tumor cells (**Figure 1**). Lactate shuttle primarily relies on monocarboxylate transporter proteins (MCTs) (7). Out of the acknowledged MCTs, MCT1-4 can be observed in different organs, contributing toprotons bonding and the bidirectional transportation of monocarboxylic acids (8). Following the attachment of the liberated proton with the MCT, lactate promptly associates with the MCT. Within the transport protein, lactate undergoes a structural change and is released along with protons from the opposite side of the membrane (9).

The combined function of MCT1-4 facilitates lactate transport between cells, being crucial for sustaining lactate homeostasis in various tissues (10). For typical tissues, MCT1 contributes a vital part in maintaining lactic acid balance by facilitating lactate transfer across the membrane in accordance with the membrane-based concentration gradient. In contrast, cells with elevated intracellular lactate levels, such as tumor cells, depend on the transporter MCT4 for the movement of lactic acid. Tumor cells regulate the expression of MCTs to maintain intracellular lactate homeostasis in order to benefit themselves and avoid harm. Certain tumor cells activate MCT4 to make use of lactate as a source of energy (11). Lactate also serves as a signaling molecule that modulates MCT expression. Lactate activates the GPR81/mTOR/HIF-1α/STAT3 pathway in pancreatic ductal adenocarcinoma cell lines, affecting the gene expression of MCT1 and MCT4 (12). In addition, glutamine can stimulate HIF-1α, thereby promoting the expression of MCT4 (13). The MCT-mediated lactate shuttle establishes intercellular connections and contributes to the cooperative metabolic interactions among various cancer cells, thereby promoting tumor initiation and progression. Lactate flowing out of tumor cells can prevent the intracellular environment from becoming more acidic, but it can cause acidification of the tumor microenvironment.

## 3 Lactic acid related pathway

### 3.1 G protein-coupled receptors pathway

The function of lactate relies on specific G protein-coupled receptors (GPRs) (14), which are located on the cell surface. They can detect extracellular molecules and trigger cellular responses (15). Classic metabolites, including lactate, possess the ability to initiate direct signal transduction via GPRs (16) (**Figure 1**). The research indicates that lactate can act as a signaling molecule via GPR81 and GPR132, which are receptors sensitive to protons (17). Among them, GPR81 exhibits high expression in various tissues such as adipose tissue, kidney, bone, and heart, mediating the influence of lactic acid on energy metabolism, lipid metabolism, inflammation, and other biological processes (35–37).

Lactate and the activation of the GPR81 signaling pathway through lactate have important implications in various aspects of tumor advancement. Research has demonstrated that GPR81 is upregulated within cancer cells in response to lactic acid signals. This indicates lactate produced by cancer cells induces GPR81, promoting a carcinogenic phenotype development (12). In addition, lactate can promote tumor growth by paracrine secretion through activating GPR81 in non-tumor cells within the TME (38). On the other hand, GPR132 is expressed in the respiratory system, digestive system and immune cells, with a particular emphasis on macrophages (39), where its expression positively correlates with M2-type macrophage presence and transition (18).

### 3.2 Lactylation modification pathway

In 2019, Zhang et al. employed high-performance liquid chromatography (HPLC)-tandem mass spectrometry (MS/MS) to detect core histones in MCF7 cells. This study discovered the mass shift observed on the lysine residues of three protein hydrolysates corresponds to the addition of a lactyl group to the ϵ-amino group of lysine (19). This research validates a novel epigenetic modification mechanism, referred to as histone lysine lactylation (Kla), that depends upon the presence of lactic acid. Histone Kla is observed to accumulate on gene promoters exposed to hypoxia, bacterial stimulation, interferon-γ (INF-γ), or lipopolysaccharide (LPS), thereby exerting an influence on gene expression. The circXRN2-Hippo pathway acts as an upstream regulator in human bladder cancer, exerting further control over tumor progression by suppressing H3K18 acetylation and inhibiting LCN2 expression (20). Instead, the oncogene BRAFV600E in undifferentiated thyroid carcinoma promotes tumor cell glycolysis, resulting in the H4K12la. This leads to dysregulation of gene transcription and the cell cycle (21). The subsequent investigations have revealed that lactylation is a prevalent PTM, occurring in both histones and non-histone proteins (22) (**Figure 1**).

It is known that p300/CREB-binding protein (CBP), which are classical histone acetyltransferases (HATs), are capable of catalyzing various acylation modifications (23, 24). An ex vivo cell-free experiment demonstrates that p300 is also capable of catalyzing the Kla reaction chemically. Multiple studies have provided evidence supporting the significant involvement of p300/CBP in regulating histone lactylation within induced pluripotent stem cells (iPSCs) and macrophages (19, 25, 26). Delactylation modification is an enzymatic process driven by histone deacetylases (HDACs): the mechanism of deacetylation was analyzed in detail through *in vitro* experiments on core histones and 18 recombinant HDACs. These findings showed that HDAC1-3 can remove Kla from histones, with HDAC3 exhibiting the most efficient erasing activity (27). Hence P300 and HDAC play a role in diverse protein modifications, thereby establishing a connection between lactylation and other PTMs. Similar to many other PTMs, Kla is regulated by the addition and removal of lactyl groups in histones theoretically (28, 29). However, the current understanding of the biochemical process of lactylation suggests that it depends on two metabolic mechanisms. Within these mechanisms, lactyl-CoA is strongly linked to enzymatic lactylation, whereas nonenzymatic lactylation involves the participation of lactyl-glutathione (LGSH) (30).

It should be emphasized that the association between lactylation modification and RNA modification is noteworthy. In the case of ocular melanoma, YTHDF2 expression is increased by Kla. This protein aids tumor progression by enhancing destruction of m6A-modified PER1 and TP53 mRNA, which it detects specifically (31). In the TIME, lactic acid increases METTL3 expression, a methyltransferase-like protein in tumor-infiltrating myeloid cells (TIM), by H3K18 modification. Meanwhile, METTL3 can be directly influenced by lactate and control the pathway via METTL3-jak1-stat3 to amplify METTL3 binding. This process makes it easier to modify target RNA with m6A, thus boosting the subsequent molecules with immunosuppressive effect production (32).

## 4 Interplay between lactate and tumor-associated immune cells

**Figure 2:**
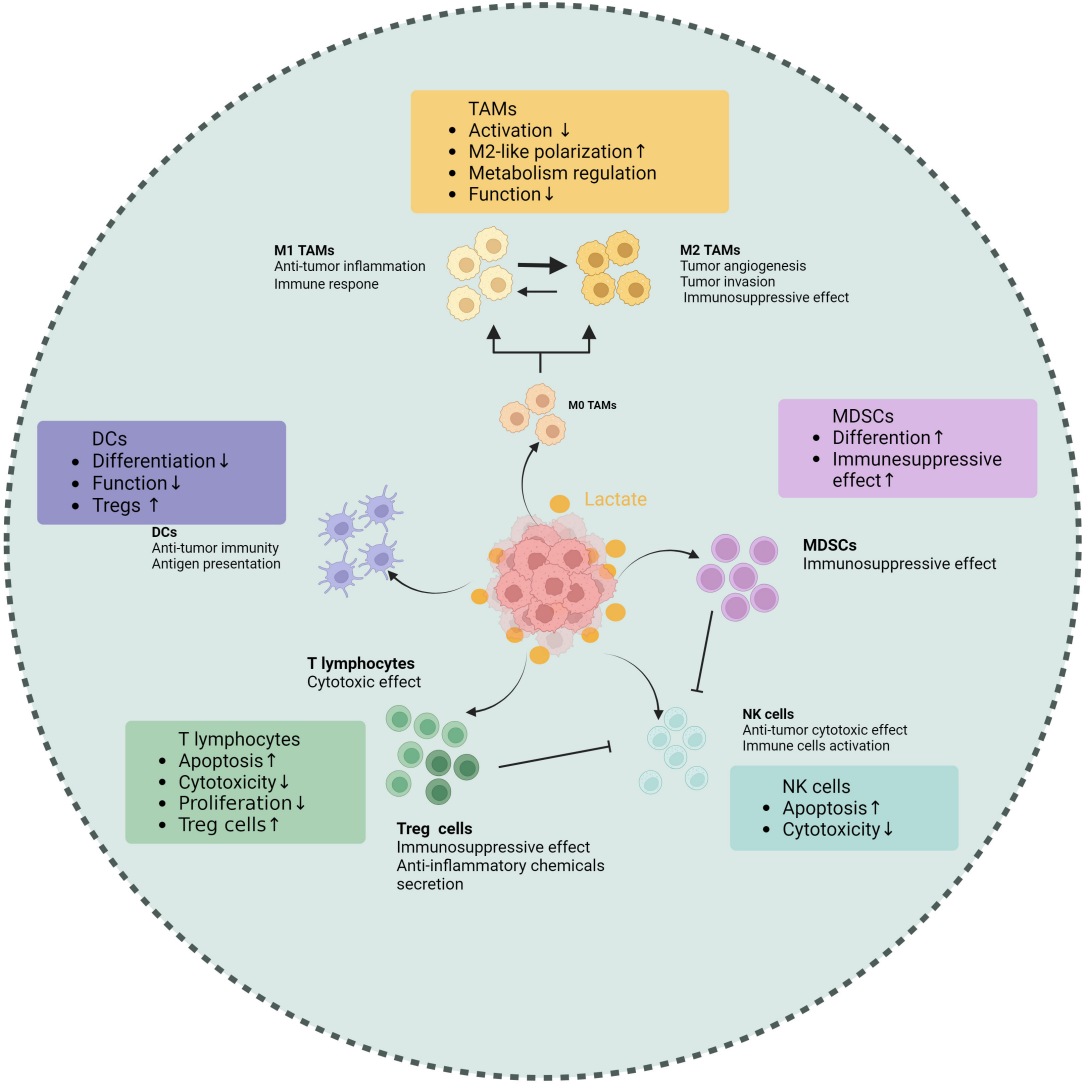
Effects of lactate on tumor-associated immune cells. Lactate makes different impacts on various immune cell populations within the TME, including tumor-associated macrophages (TAMs), T cells, natural killer (NK) cells, dendritic cells (DCs), and myeloid-derived suppressor cells (MDSCs). Elevated lactate levels are shown to promote the polarization of TAMs towards an immunosuppressive phenotype, enhancing their ability to support tumor growth. In T cells, lactate impairs proliferation and cytokine production, leading to reduced antitumor activity. NK cell function is also inhibited by lactate, which diminishes their cytotoxic potential. Conversely, lactate enhances the immunosuppressive properties of MDSCs, facilitating their accumulation in the tumor microenvironment. Dendritic cells exhibit altered maturation and function in the presence of lactate, impacting their ability to activate T cells. These findings underscore the role of lactate as a critical metabolic regulator of immune responses in tumors, highlighting its potential as a therapeutic target. (Created with BioRender.com).

One of the key regulating mechanisms of the TIME is lactate within the TME, which has abilities to affect various tumor-associated immune cells to exert immune suppression (**Table 1**). First, lactate is able to exert an influence on the metabolism and cellular respiration of immune cells themselves. Furthermore, the acidifying effect of lactate can lead to a reduction in immune cell function or modulation of downstream signal transduction pathways (**Figure 2**). Additionally, lactate has the ability to interfere with the identification and lethal activities of immune cells by either suppressing or enhancing the expression of ligands or receptors on both immune and tumor cells (**Figure 2**).

**Table 1:** Effects of lactate on tumor-associated immune cells.

Cell type	Effect	Mechansim
Macrophage	Activation↓	inhibit Yes1-associated transcription factor and NF-κB
	M2-like polarization↑	promote ERK/STAT3 signaling pathway
		enhance the stability of MCT/HIF1α
		increase the intercellular ROS and activate Nrf2
		activate GPR132
		lactylation modification
	Metabolism regulation	shift from OXPHOS to glycolysis metabolism
	Function↓	activate the Ap-α/Elk-1 axis
		upregulate PD-L1 expression
		regulate the release of HMGB1
T lymphocyte	Cytotoxicity↓	inhibit lactate efflux
		alter the balance of the TCA cycle
		inhibit p38 and JNK–JUN signaling
		inhibit NFAT and IFN-γ production
		reduce cholesterol synthesis and IFN-γ release in iNKT cells
	Proliferation↓	regulate the NAD(H) oxidation-reduction state
	Treg cells↑	FOXP3-mediated repression of MYC
		upregulate the expression of CD25
		modulate lactylation of MOESIN, improve MOESIN interaction with TGFβ/SMAD3 signaling
		promote NFAT1 translocation into the nucleus, enhance the expression of PD-1
	Apoptosis↑	promote apoptosis of immature T cells
NK cells	Cytotoxicity↓	inhibit NFAT and IFN-γ production
		acidize pH environment
		inhibit the expression of NKp46
		inhibit mTOR signaling
	Apoptosis↑	inhibit lactate efflux
DCs	Differentiation↓	affect the differentiation of monocytes into DC
		alter antigen expression and decrease IL-12
	Function↓	accelerate antigen degradation and hinder cross-presentation
		activate GPR81
		inhibit the induction of IFN-α and IFN-γ in pDCs
	Treg cells ↑	promotes the metabolism of tryptophan and L-kynurenine
MDSCs	Differention↑	activate Notch
	Immunesuppressive effect↑	GM-CSF and IL-6↑
		activate GPR81/mTOR/HIF-1α/STAT3
		induce lactylation-driven METTL3-mediated RNA m6A modification

↑: enhance.↓: disminish.

### 4.1 Tumor-associated macrophage

It is not surprising that lactate can influence tumor-associated macrophages (TAMs), which are the most abundant immune cell population found in TIME. In terms of its mechanism, the GPR81a binds to lactate and exerts inhibitory effects on the activation of Yes1-associated transcription factor and NF-κB, thereby effectively suppressing macrophage activation (33). The macrophages in TME can generally be classified into M1 type (classically activated macrophages) and M2 type (alternatively activated macrophages). The presence of M1 macrophages in the TME inhibits tumor growth and is linked to a better prognosis in various cancers. In contrast, the M2 macrophages promote tumor initiation and progression (34, 40, 41). As a biological process, “macrophage polarization” is controlled by certain microenvironmental cues that govern the transition between M1 and M2 macrophages. Elevated lactate concentration is a key driving factor for TAM polarization (42). Initiation of STAT3 and ERK1/2 pathways in the tumor microenvironment (TME) has been shown in earlier research to induce M2 polarization in macrophages. Research that followed identified lactic acid as an ERK/STAT3 pathway promoter (43). Lactate enhances the stability of HIF-1α and MCT to increase the expression of Vascular Endothelial Growth Factors (VEGF) and arginine in TAMs, ultimately promoting their polarization towards an M2 type (44). The presence of lactate may potentially cause a rise in intracellular ROS levels, thereby activating Nrf2 in macrophages and promoting their transition into the M2 macrophage (45). In the breast cancer and Lewis lung cancer models, the lactate signal within the TME can activate GPR132, thereby promoting the M2 phenotype (46–48). The following discovery of histone lactylation revealed a novel mechanism whereby lactylation of histone arginine residues can regulate TAM polarization by increasing the ARG1 expression or other TAM-related genes (19). In summary, lactate-induced M2-type TAMs within the TME undergo polarization and facilitate immune evasion, thereby contributing to the sustenance of tumor progression and viability.

Additionally, lactate has the ability to control TAM metabolism, switching them from OXPHOS to glycolysis. In the long run, this helps tumor growth by increasing the release of lactate (49). Lactate has the potential to influence macrophages themselves function. The killing action of macrophages on tumors is effectively inhibited when CD47 on tumor cells binds with SIRP-α on macrophages. In colorectal cancer, lactate induces activation of the Ap-α/Elk-1 pathway in TAMs, which raises the expression level of SIRP-α, further suppressing tumor immune response (50). Additionally, lactate can upregulate the PD-L1 expression in TAMs, thereby facilitating immune evasion within the TME (51). Recent investigations have demonstrated that damage-associated molecular patterns (DAMPs), including high mobility group box protein 1 (HMGB1), which is associated with lactate and lactylatio (52), exert pivotal roles in triggering and perpetuating inflammation, thereby compromising immune cells and fostering tumorigenesis (53, 54).

### 4.2 T lymphocyte

T lymphocytes serve as the main force in the immune system by exerting their cytotoxic effects on tumor cells. The high level of lactate has been demonstrated to negatively impact almost every aspect of T lymphocyte function (55). First, activated T cells require glycolytic metabolism and must secrete endogenous lactic acid to prevent cellular acidification. However, lactate accumulation inhibits T cells from releasing lactate, leading to disruptions in their metabolism and function. In addition, lactate can change the balance of the TCA cycle, finally impairing the cytotoxic T lymphocyte (CTL) function (56). Lactate accumulation within the TME may block p38 and JNK/c-Jun signal transduction to impair CTL function (57). Lactate suppresses nuclear factor of activated T cells (NFAT) expression in T cells and natural killer (NK) cells, leading to a decreased level of IFN-γ. This hinders the capacity of T cells and NK cells to conduct immune surveillance (58). Regarding proliferation, lactate regulates the NAD(H) oxidation-reduction state to limit T cell proliferation. Lactic acid can reduce NAD+ to NADH and alter NAD+-dependent enzyme reactions, thereby reducing the generation of intermediate glycolysis products vital to T cell proliferation and achieving limitation on their proliferation (59).

Regulatory T (Treg) cells, a specific subgroup of CD4+ T cells, contribute to tumor immune tolerance by inhibiting the proliferation and promotion of immune cells and secreting anti-inflammatory chemicals. Compared to other T cells, Treg cells exhibit different characteristics in the acidic TME. As a specific molecule of Tregs, FoxP3 maintains Tregs’ OXPHOS metabolism and provides metabolic advantages by inhibiting the c-Myc signaling pathway in a high glycolytic microenvironment (60). Lactate can enhance Treg infiltration in tumors along with upregulate CD25 expression, a surface marker associated with Treg activation (61). From a mechanistic standpoint, lactate may regulate Treg cell activity by facilitating MOESIN lactylation and modulating TGF-β signaling transduction, thereby contributing to their maturation and differentiation processes (62). Furthermore, Tregs enhance the functionality of PD-1 by actively absorbing lactate through the MCT1, promoting NFAT1 translocation into the cell nucleus (63).

Lactic acid not only affects common types of T cells but also promotes apoptosis in immature T cells and decreases cholesterol synthesis and IFN-γ release in invariant natural killer T (iNKT) cells, thereby influencing tumor immunity (64, 65).

### 4.3 Natural killer cells

NK cells serve as the primary defense by releasing molecules, including cytokines and granule enzymes, which facilitate the elimination of cancer cells. Moreover, they possess the ability to activate additional immune cells, thus augmenting the overall immune reaction.

Study indicates that the acidic tumor microenvironment negatively affects the functionality of NK cells (66, 67). Further experiments have revealed that the spontaneous release of LDH, a key enzyme in lactate production, was found to be associated with NK cell dysfunction (68). The lactate derived from tumors can inhibit NKp46 expression, resulting in suppression of cytotoxicity in NK cells. This decline in NK cell cytotoxicity is often associated with decreased perforin and granzyme levels (69). In addition, lactate can impair NKT cell function by interfering with the mTOR signaling pathway (70). In terms of cell homeostasis, colorectal cancer cells that metastasize to the liver produce a large amount of lactate when faced with NK cells with strong cytotoxicity in the liver. This lactate production lowers the pH value in the TME, thereby preventing NK cells from effectively removing lactate from the cytoplasm through concentration gradients. As a result, mitochondrial stress and cell apoptosis occur (71).

### 4.4 Dendritic cells

Dendritic cells (DCs) play a crucial role in anti-tumor immunity through their function in antigen presentation. Research indicates that lactic acid in the TME can affect DC and block its differentiation (72). Subsequently, Gottfried et al. developed multicellular tumor spheroids (MCTS) to facilitate the infiltration of monocytes and immune cells into MCTS comprising multiple tumor cells derived from diverse origins. They found that tumor-derived lactate was a potent modulator of human monocytes. Lactate can not only inhibit monocytes differentiating into DCs, thereby impairing antigen presentation, but also interfere with the migration of monocytes into MCTS (73). The phenotype of DCs is significantly influenced by lactate derived from tumors. The addition of lactate *in vitro* can induce alterations in antigen expression and a decrease in IL-12 secretion, similar to the phenotype change of tumor-associated DCs observed in co-cultures of melanoma cancer, and reducing lactate level can restore the normal phenotype of DCs (74). In lung cancer, lactate changes the adaptability of DCs by making it harder for cross-presenting to happen and making it easier for antigens to disappear (75). According to a breast cancer report, lactate activates GPR81 on DCs, which hinders the display of tumor-specific antigens to additional tumor-associated immune cells (38). The presence of lactate can impede the activation of IFN-α and IFN-γ in plasmacytoid dendritic cells (pDCs), which constitute a subset of dendritic cells and are recognized as the most effective producers of IFN-α in humans. thereby compromising the effectiveness of the immune response against tumors. Additionally, lactic acid promotes the metabolism of tryptophan and L-kynurenine generation in pDCs, facilitating the development of significant immunosuppressive immune cell groups in the tumor microenvironment, specifically FoxP3+CD4+Tregs (76).

### 4.5 Myeloid-derived suppressor cells

Myeloid-derived suppressor cells (MDSCs) refer to a diverse group of cells originating from the bone marrow that possess an extraordinary capacity to effectively inhibit immune reactions.

After the activation of the Notch/RBP-J pathway, the downstream molecule HES1 reduces the expression of MCT2, ultimately lowering the intracellular lactic acid concentration, which in turn affects the differentiation of MDSCs and the maturation of TAMs (77). As early as 2013, it was discovered that lactic acid derived from tumors increases the frequency of MDSCs (69). Subsequent research indicates that lactate attracts MDSCs and controls their maturation, thus influencing tumor immunity and the advancement of tumors (77, 78).

Another study on pancreatic cancer has shown that lactate activates MDSCs through the GPR81/mTOR/HIF-1α/STAT3 pathway, thereby enhancing the immunosuppressive effects (79). Lactic acid has the potential to enhance the immunosuppressive capabilities of TIMs through epigenetic mechanisms. METTL3’s zinc finger domain contains two lactylation modification sites, which are associated with RNA m6A modification within MDSCs (32).

## 5 Clinical relevance of lactic acid

In the in-depth study of the unique metabolic patterns and immune response in tumors, scientists have discovered numerous therapeutic strategies targeting tumor metabolism and immunity. Lactate suppresses immune cell activity, diminishing their capacity to attack malignant cells and creating favorable conditions for tumor growth and spread. Currently, numerous clinical studies are being conducted to explore the significance of lactate in clinical practice as a specific metabolite. Therefore, intervening in lactate metabolism pathways holds promise for altering the tumor microenvironment and enhancing immunotherapy effectiveness.

### 5.1 Prognostic markers

Under normal circumstances, serum lactic acid concentration typically ranges from 1.5-3.0 mM (80). Due to the Warburg effect, lactate concentration in tumors can reach a range of 10-30 mM and even grow up to 50 mM within the necrotic core of the tumor (81). Indeed, elevated lactic acid levels are considered unfavorable prognostic indicators for various cancers, including cervical cancer (80), breast cancer (82), head and neck cancer (83), and non-small cell lung cancer (84). Hence, the assessment of patient prognosis and treatment selection can be facilitated by examining lactate and related metabolite levels (**Table 2**). New advancements in technology, such as Magnetic Resonance Spectroscopy (MRS) and Hyperpolarized 13C-MRI (HP 13C-MRI),are reforming the measurement approach for lactic acid and enhancing the practicality of utilizing the level as a cancer diagnostic tool. Current investigations into lactate imaging as a diagnostic biomarker for tumors encompass such as NCT01881386 (observing alterations in lactate concentration following treatment through magnetic MRS), NCT04584827 (examining the impact of lactate concentration death and morbidity in patients undergoing intracranial tumor surgery under general anesthesia), NCT03129776 (using HP 13C-MRI to determine radiation-resistant areas in neck tumors and guide radiotherapy), and NCT03531307 (investigating the association between lactic acid levels and tumor proliferation marker Ki67 in brain tumor patients).

**Table 2:** Clinical trials of lactate as a prognostic marker.

Trial topices	Tumor type	ClinicalTrials. Gov ID	Study Type	Research Phase	Status	Reasons for failure
Lactate Imaging as a Tumour Biomarker	Lymphoma, Metastatic Colorectal, Primary Brain Tumours and Cerebral Lymphoma	NCT01881386	Observational	\	Completed	\
Evaluation of Lactate in Patients Undergoing Glial and Non Glial Mass Surgery With Craniotomy	Intracranial tumor	NCT04584827	Observational	\	Completed	\
Hyperpolarized 13C MR Imaging of Lactate in Patients With Locally Advanced Cervical Cancer (LACC) Cervical Cancer	Uterine Cervical Neoplasms	NCT03129776	Interventional	Phase I	Terminated	Lack of participants
Lactate Levels Correlates With Ki-67 in Brain Tumor Surgery	Brain tumors	NCT03531307	Observational	\	Completed	\
Development of Magnetic Resonance Spectroscopy (MRS) Biomarkers of Tumor Metabolism (MK-0000-145) (MRS Tumor)	Glioma	NCT01138813	Observational	\	Completed	\
Hyperpolarized Carbon C 13 Pyruvate Magnetic Resonance Spectroscopic Imaging in Detecting Lactate and Bicarbonate in Participants With Central Nervous System Tumors	Central Nervous System Tumors	NCT03565367	Interventional	Phase I	Completed	\
Hyperpolarized 13C Pyruvate MRI Scan in Predicting Tumor Aggressiveness in Patients With Renal Tumors	Renal Tumor	NCT04258462	Interventional	Phase II	Recruiting	\
Role of Hyperpolarized 13C-Pyruvate MR Spectroscopy in Patients With Intracranial Metastasis Treated With (SRS)	Intracranial Metastasis	NCT03324360	Interventional	Phase I	Recruiting	\
Hyperpolarized Carbon-13 Imaging of Metastatic Prostate Cancer	Prostate Cancer	NCT02844647	Interventional	Phase I	Terminated	Lack of participants

Clinical data comes from ClinicalTrials. gov (https://www.clinicaltrials.gov/).

Instead of measuring lactate level directly, specific proteins associated with lactate metabolism can also be utilized as indicators for tumor progression. For example, increased levels of LDH have been linked to the presence of aggressive clinical pathological characteristics in pancreatic cancer and an unfavorable prognosis in mesothelioma and lung cancer (85). MCTs can serve as biomarkers and potential therapeutic targets for colorectal cancer (86). Additionally, the level of lactylation in gastric cancer tissue can serve as an indicator of tumor immune evasion and advancement (87).

### 5.2 Therapeutic target

Given the crucial involvement of lactate in tumor progression, interventions aimed at lactate could potentially impede tumor proliferation and hinder metastasis (**Table 3**). Due to the increased expression of LDHA in cancer and its primary role in lactate production position, LDHA is a potential candidate for cancer treatment. In reality, numerous studies have shown that inhibiting LDHA can effectively impede the proliferation and metastasis (88–91). Various compounds, such as Gossypol (also referred to as AT-101) and its derivative FX-11, along with galloflavin, have been identified as potential inhibitors of LDHA with promising anti-tumor properties (92). Due to its ability to inhibit LDHA, vitamin C may be considered an effective therapy for stress-related breast cancer (93). Although LDHA has shown potential in cancer therapy, its inhibition can cause a variety of off-target effects. For instance, inhibition of LDHA leads to an increased intracellular pyruvate and NADH level, which drives extracellular matrix (ECM) remodeling and ultimately strengthens collagen protein durability and promotes the progression of breast cancer (94). Targeting LDHB could also offer a potential approach for cancer therapy. LDHB is essential for enhancing lysosomal function and tumor autophagy. When LDHB is silenced, selective inhibitory effects on cancer proliferation have been confirmed (95).

**Table 3:** Clinical trials of lactate as a therapeutic target.

Target	Drugs	Tumor type	ClinicalTrials. Gov ID	Research Phase	Status	Reasons for failure
LDH	Gossypol(AT-101)	Brain and Central Nervous System Tumors	NCT00390403	Phase I	Completed	\
		Breast cancer	NCT06133088	Phase II	Recruiting	\
		B-cell Non Hodgkin Lymphoma	NCT05338931	Phase I/II	Recruiting	\
PDH	Dichloroacetate	Glioblastoma	NCT01111097	Phase I	Completed	\
		Squamous Cell Carcinoma of the Head and Neck	NCT01386632	Phase II	Completed	\
		Glioblastoma Multiforme	NCT05120284	Phase II	Recruiting	\
HK	2-deoxy-D3-glucose (2DG)	Lung cancer, Breast cancer, Pancreatic cancer, Head and Neck cancer, Gastric cancer	NCT00096707	Phase I	Completed	\
		Prostate cancer	NCT00633087	Phase I/II	Terminated	Slow accrual
PK	TLN-232	Melanoma	NCT00735332	Phase II	Terminated	License termination
PI3K/AKT/mTOR	Nab-rapamycin (ABI-009)	Non-muscle Invasive Bladder Cancer (NMIBC)	NCT02009332	Phase I/II	Completed	\
		Desmoid Tumor, Non Small Cell Lung Cancer, Colorectal Cancer, ect	NCT03190174	Phase I/II	Completed	\
	Everolimus	Breast Cancer	NCT00863655	Phase III	Completed	\
		Endometrial Cancer	NCT01068249	Phase II	active	\
		Metastatic Renal Cell Carcinoma	NCT03095040	Phase III	unkown	\
	AZD5363	Prostate Cancer	NCT04087174	Phase I	Completed	\
		Solid and Hematological Malignancies	NCT04944771	Phase I	Completed	\
Lactate transporters	Diclofenac	Basal cell carcinoma	NCT01935531	Phase IV	Completed	\
	AZD3965	Diffuse Large B Cell Lymphoma, Burkitt Lymphoma	NCT01791595	Phase I	Completed	\
	Fluvastatin	Glioma	NCT02115074	Phase I	Completed	\
Lactylation	EP31670	Castrate Resistant Prostate Cancer, NUT Carcinoma	NCT05488548	Phase I	Recruiting	\
	CCS1477	Metastatic CastrationResistant Prostate Cancer, Metastatic Breast Cancer, Nonsmall Cell Lung Cancer, Advanced Solid Tumors	NCT03568656	Phase I/II	Recruiting	\

Clinical data comes from ClinicalTrials. gov (https://www.clinicaltrials.gov/).

Except for the previously mentioned LDH, other glycolytic enzymes such as PDH, hexokinase II (HK2), and pyruvate kinase isozyme M2 (PKM2) can also be targeted to decrease lactate production (96). Additionally, disrupting lactate-producing signaling pathways like the PI3K/AKT/mTOR could be a potential approach (97). Direct consumption of lactate is an alternative approach. Lactic acid oxidase (LOX) can oxidize the lactate secreted by tumors into pyruvate and hydrogen peroxide (H_2_O_2_), thereby addressing lactate-induced drug resistance (98).

For lactate transporters, one possible antitumor approach could involve the inhibition of MCT1 and MCT4 activity or the reduction of their presence (99). Syrosingopine has been demonstrated as a potent dual inhibitor of MCT1 and MCT4 (100) making tumor cells more susceptible to metformin, thereby boosting the anticancer effects of metformin. These results highlight the possibility of syrosingopine as a supplementary treatment in upcoming clinical agents (101). The cell surface localization of MCT1 and MCT4 is dependent on the CD147 (102). For this reason, CD147 was presented as a prospective option to increase the efficacy of cancer treatment and intervention in MCT membrane integration. However, MCTs may also serve as transport proteins for specific medicine, like the potential anticancer agent 3-bromopyruvate (3-BP) (103). Consequently, if used in combination, there might be potential impediments to the pharmacological effects.

The observation of lactylation has greatly broadened the paradigm of lactic acid-related therapies beyond traditional targets. Several studies have been conducted focusing on histone lactylation. Demethylzellal (DML) has been shown to efficiently increase the effectiveness of chemotherapy treatments by preventing H3Kla in stem cells from liver cancer (104). Royal jelly acid (RJA) has been found to inhibit the progression of hepatocellular carcinoma (HCC) by suppressing H3K9la and H3K14la (105). Clinical studies are now under progress to assess the effectiveness of p300 inhibitors, including EP3160 and CCS1477, in directly inhibiting the lactylation process.

Due to the strong correlation between lactate and tumor immunity, a potential strategy for tumor treatment could involve combining lactate-targeted cancer therapy with immunotherapy. For example, by reducing lactate production while simultaneously administering PD-1 inhibitors, treatment efficacy may be enhanced.

## 6 Conclusion and perspectives

In this review, we convey a summary of the unique lactate metabolism mechanism in tumors and the formation of acidic TME, as well as summarize the signaling pathways related to lactate. We emphasize the impact that lactic acid has on serval tumor-associated immune cells. Furthermore, we debate the prospective application of lactate as a tumor biomarker and clinical anticancer target.

Lactate is classified into three types according to the arrangement of carbon atoms, including D-lactate, L-lactate, and racemic DL-lactate. Present studies primarily focus on L-lactate in the realm of lactate research. In contrast, D-lactate, as an enantiomer of L-lactate, is typically present in negligible amounts within human tissues and thus remains undetectable in the bloodstream under normal physiological circumstances (106). Research has shown the regulatory function of D-lactate in cellular metabolism, antioxidant capacity, and energy generation, along with its correlation to disease (107–109). Moreover, D-lactate has been associated with the control of tumor growth and immunity, contributing to the development of various cancers such as esophageal cancer, prostate cancer, hepatocellular carcinoma and renal cell carcinoma (110–113). However, there are still significant gaps in comprehension involving the role of D-lactate and its impact on tumors. Therefore, further investigation in this field is necessary and meaningful.

Exploring lactylation is expected to emerge as a promising avenue for investigating the regulation of cell function in the TME and understanding mechanisms underlying tumor progression. Additionally, identifying enzymes or genes associated with lactylation modification could offer novel therapeutic targets. Nevertheless, there still exist a multitude of unresolved enigmas in this domain that merit further investigation. First, besides histone lactylation, what impact do other non-histone lactylations associated with KLA sites have on tumor angiogenesis, progression, invasion, and immune response? Secondly, what are the main factors of specialization and discrimination in the p300/CBP function concerning the function selectivity of acyl modifications? All in all, how does an indiscriminate HAT particularly facilitate lactylation? Research regarding Kla has identified additional forms of acylation occurring concurrently with lactylation on identical lysine residues of the same proteins, indicating the possibility of interplay between these modifications (114). So, there is still an unanswered question regarding the connections between lactylation and other PTMs. Additionally, it is crucial to determine the specific mechanism by which histone modifications interact with RNA modifications. By addressing these inquiries, we can enhance our comprehension of lactylation’s contribution to tumor development and subsequently propose more precise and promising treatment alternatives.

In relation to lactate and tumor immunity, it is commonly believed that lactate may potentially compromise the effectiveness of tumor immunity and facilitate immune evasion. However, lactate has a dual effect on T cells; in addition to inhibiting the function of T cells, the research conducted by Ing Wen revealed the potential of sodium lactate therapy in suppressing tumor development in living organisms, relying on the presence of T cells (115). Furthermore, CD8+ T cells have the capability to utilize lactate as a supplier for power and vital components, which improves their metabolic function and promotes an immune response against tumors (116). These offer a fresh outlook on the interactions between tumor cells and immune cells facilitated via lactic acid.
